# Inflammation-driven mechanisms in endometrial cancer: pathways from inflammatory microenvironment remodeling to immune escape

**DOI:** 10.3389/fimmu.2025.1689114

**Published:** 2025-11-26

**Authors:** Zhaoping Tan, Binyue Sheng, Lu Chen, Hong Dong, Yaqin Deng, Yunyun Li, Cong Liu, Han Wang, Zi Yang, Ting Xie, Yanming Huang

**Affiliations:** Department of Gynecology, Maternal and Child Health Hospital of Hubei Province, Tongji Medical College, Huazhong University of Science and Technology, Wuhan, China

**Keywords:** endometrial cancer (EC), inflammatory microenvironment (IME), immune escape, immune checkpoint blockade, pembrolizumab–lenvatinib

## Abstract

The progression of endometrial cancer (EC) is significantly affected by the inflammatory microenvironment (IME), which is essential for facilitating immune evasion and developing resistance to therapeutic interventions. Components that promote immune suppression, such as regulatory T cells (Tregs), macrophages associated with tumors (TAMs), cytokines like interleukin-10 (IL-10) and transforming growth factors-beta (TGF-β), are crucial in establishing a favorable microenvironment for tumor growth. TAMs with a M2-like phenotype promote angiogenesis and inhibit antitumor immunity through the secretion pro-tumorigenic factor. Further, metabolic shifts in the extracellular matrix and structural modifications of the extracellular matrix (ECM) inhibit the infiltration of cytotoxic T lymphocytes (CTLs), thereby strengthening mechanisms of immune evasion. Inflammatory signaling pathways, such as interleukin-6/janus kinase/signal transducer and activator of transcription 3 (IL-6/JAK/STAT3) and NF-κB/tumor necrosis factor-alpha (TNF-α/NF-κB), also stimulate the expression immune checkpoint molecules, such as programmed cell death protein 1 (PD-1). Novel interventions aimed at modulating immune checkpoints, inhibiting TGF-β signaling, and altering metabolic circuits are under investigation and offer potential to counteract immune suppression and enhance therapeutic success. Nevertheless, significant obstacles remain, including intratumoral heterogeneity, fluctuating immune dynamics, and the absence of dependable biomarkers. Advancements in single-cell analysis and spatial transcriptomics are anticipated to unveil actionable molecular patterns and support the development of individualized strategies to interrupt immune evasion and therapeutic resistance in EC. These advances offer promise for personalized immunotherapy approaches that could significantly improve outcomes in endometrial cancer patients.

## 1 Introduction

Endometrial carcinoma (EC) constitutes nearly 90% of uterine cancers and stands as the most frequently diagnosed gynecologic malignancy in industrialized nations, posing a serious challenge to women’s health and overall well-being (1, 2). Its global incidence is steadily increasing and is strongly linked to various predisposing factors, such as prolonged exposure to estrogen without sufficient progesterone, excess body weight, insulin resistance, and elevated blood pressure (3, 4). Among these, persistent hormonal dysregulation—most notably sustained estrogen dominance without progesterone counterbalance—drives endometrial tissue proliferation and fosters malignant development (5). EC is closely related to metabolic disturbances, such as obesity and diabetes type 2, which play a pathogenic role. Adipose tissue is an active endocrine system in overweight individuals. It secretes proinflammatory cytokines, such as tumor necrosis factor-alpha (TNF-α) and interleukin-6(IL-6), that promote a chronic inflammatory condition that facilitates the development of endometrial cancer (6, 7). Moreover, hyperglycemia and insulin resistance in diabetic patients further exacerbate this proinflammatory milieu, indirectly promoting tumor development (8).

Recent evidence highlights that inflammation and immune dysregulation are not merely coincidental but serve as active drivers of EC initiation and progression. This mini review concentrates on the inflammatory microenvironment (IME), which is the inflammatory element within the wider tumor microenvironment (TME), including immune cells, inflammatory agents, and their communication networks. The tumor microenvironment (TME) encompasses every cellular and molecular element encircling the tumor, with the tumor immune microenvironment (TIME) specifically denoting immune cell groups, whereas the IME symbolizes the confluence of inflammatory and immune mechanisms propelling tumor development.

In the context of EC, the dynamic alterations occurring within the IME, along with the development of immune evasion tactics by tumor cells, play a pivotal role in facilitating malignant progression. This progression encompasses increased cellular proliferation, invasion, and metastasis. The IME is composed of a variety cellular and molecular elements, including tumor-associated macrophages (TAMs), Th cells, regulatory T cells (Tregs), natural killer (NK) cells and cytokines, including transforming growth factor beta (TGF-β) and vascular endothelial-growth factor (VEGF) (9). These elements interact through complex signaling networks that collectively modulate tumor behavior and immune escape (10).

At the molecular level, immune escape in EC involves several mechanisms that impair antitumor immunity. Endometrial cancer cells utilize several immune escape strategies, including enhanced expression of checkpoint regulators such as programmed cell death protein 1 (PD-1) and PD-L1, impaired antigen presentation due to reduced major histocompatibility complex class I (MHC I) expression, and enzymatic activation of immunosuppressive mediators like indoleamine 2,3-dioxygenase 1 (IDO1). Together, these mechanisms suppress immune recognition and dampen the cytotoxic function of T cells (11). The resulting immunosuppressive state, in concert with ongoing inflammatory signaling, supports an environment conducive to tumor progression.

**Figure 1:**
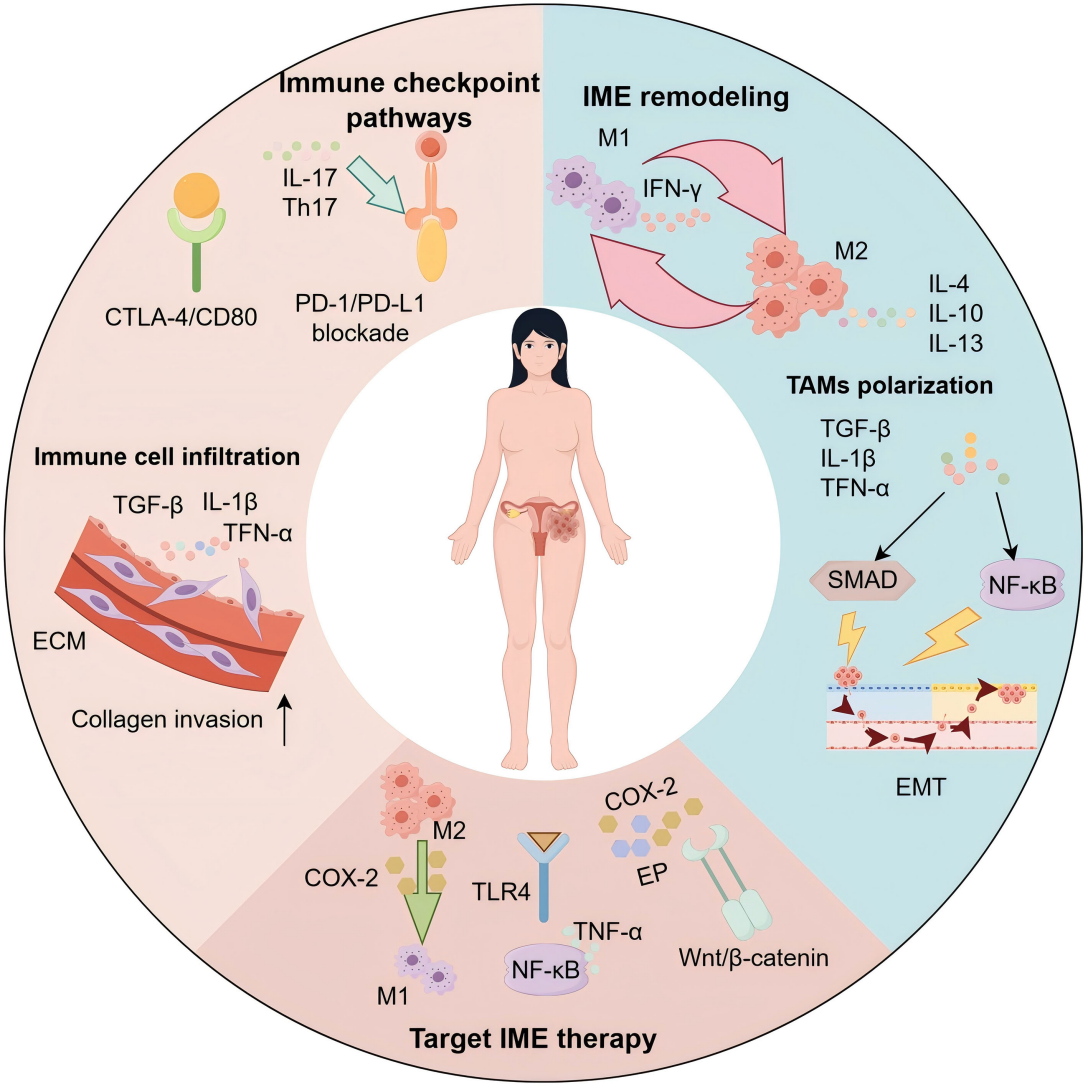
Inflammatory microenvironment of endometrial cancer: mechanisms and therapeutic targets. Schematic of the endometrial cancer (EC) inflammatory microenvironment (IME). Immune-cell infiltration and extracellular matrix (ECM) remodeling driven by TGF-β, IL-1β, and TNF-α facilitate collagen invasion. Immune-checkpoint signaling (PD-1/PD-L1; CTLA-4/CD80) and Th17/IL-17 sustain protumor inflammation. Tumor-associated macrophages (TAMs) polarize from M1 (IFN-γ–associated) to M2 states under IL-4/IL-10/IL-13. Activation of NF-κB and SMAD pathways promotes epithelial–mesenchymal transition (EMT) and immune escape. Targetable nodes highlighted include the COX-2/PGE_2_/EP axis, TLR4–NF-κB, and Wnt/β-catenin, supporting combinations with checkpoint blockade and strategies that repolarize TAMs toward M1.

This review seeks to clarify the essential cellular and molecular mechanisms by which inflammation alters the tumor microenvironment and promotes immune evasion in endometrial cancer. Additionally, we investigate how these interconnected processes contribute to tumor advancement and the development of resistance to therapies. Ultimately, we put forth a comprehensive conceptual framework that could shape future research trajectories and assist in the formulation of innovative therapeutic approaches for the tailored treatment of endometrial cancer (12). **Figure 1** provides an overview of the inflammatory microenvironment (IME) in endometrial cancer, depicting immune-cell infiltration and ECM remodeling, macrophage polarization (M1/M2), canonical checkpoint signaling (PD-1/PD-L1, CTLA-4/CD80), and representative IME-targeted strategies.

## 2 Inflammatory microenvironment remodeling in EC

### 2.1 Cellular and soluble components of the IME

The IME in EC represents a complex and evolving network of immune cells and secreted factors that promote malignant progression, suppress immune surveillance, and contribute to therapeutic resistance (13). Critical cellular players including tumor-associated macrophages (TAMs), Tregs, Th, NK cells, and neutrophils actively reshape the IME by releasing cytokines, including IL-1β, TNF-α, TGF-β, and VEGF (13, 14). In EC, TAMs are predominantly M2-polarized, producing interleukin-10 (IL-10) and TGF-β to promote immunosuppression and angiogenesis (14). Tregs suppress effector T cells, diminishing antitumor immunity and correlating with poor clinical outcomes (14). Among Th subsets, Th1 cells mediate cytotoxicity via interferon-gamma (IFN-γ), while Th2 and Th17 cells, through IL-4, IL-13, and IL-17, support tumor progression (13). Despite their inherent cytotoxicity, NK cell function is frequently impaired in EC (13). Neutrophils recruited to tumor sites can differentiate into tumor-associated neutrophils (TANs), which secrete matrix metalloproteinase-9 (MMP-9) and VEGF, promoting extracellular matrix degradation, angiogenesis, and metastasis (15, 16). While Th subsets are discussed broadly, the role of Th17/IL-17 in EC remains controversial, with both pro- and anti-tumor effects reported across studies; we revisit this debate in Section 3.1 when considering checkpoint regulation (17, 18).

The function of Th17/IL-17 in endothelial cells is contingent on the particular situation and remains a topic of discussion (18). In the early phases of illness and for microsatellite unstable (MSI-H) tumors, Th17 cells are capable of inhibiting tumor expansion by enhancing antigen presentation and luring cytotoxic lymphocytes (19). Conversely, Th17 cells in advanced endothelial cells, marked by increased levels of IL-6 and TGF-β, promote angiogenesis, EMT, and immunosuppression by activating NF-κB, which is facilitated by IL-17 (20). Collectively, these immune populations and their cytokine milieu establish an immunosuppressive feedback loop that facilitates EC progression and immune escape.

### 2.2 Pathways driving inflammatory microenvironmental remodeling

#### 2.2.1 Polarization of TAMs

TAMs exhibit functional heterogeneity in EC, oscillating between antitumor M1 and protumor M2 phenotypes (21). M1-like TAMs are induced by microbial products or IFN-γ, enhancing antigen presentation and cytotoxicity via TNF-α, IL-12, and IL-1β secretion (22–24). Conversely, M2-like TAMs are driven by IL-4, IL-13, and IL-10, and promote tumor progression by releasing immunosuppressive factors (PGE2, TGF-β, IL-10), and pro-angiogenic mediators (FGF, PDGF, VEGF) (14). Metabolic cues such as lactate accumulation and hypoxia stabilize HIF-1α, reinforcing M2 polarization and sustaining immunosuppressive functions (24, 25). These TAMs impair antitumor immunity, remodel the extracellular matrix, and drive EC progression. Modulating TAM plasticity may thus offer a therapeutic avenue to reprogram the immune microenvironment (26).

#### 2.2.2 ECM remodeling and stromal crosstalk

In the context of the tumor microenvironment, the extracellular matrix (ECM) functions as an essential structural framework and a signaling hub that modulates epithelial–mesenchymal transition, thereby influencing the biological characteristics EC. Inflammatory cues trigger the secretion of chemokines and cytokines, which subsequently alter ECM architecture and modulate the activity of matrix-remodeling enzymes (27). For example, TNF-α, TGF-β, and IL-1β activate NF-κB and SMAD signaling to promote collagen synthesis and matrix metalloproteinase (MMP) expression, thereby altering ECM properties and facilitating invasion (28). TGF-β facilitates the transformation of resident fibroblasts into cancer-associated fibroblasts (CAFs). These CAFs significantly contribute to the remodeling of the stromal environment by synthesizing extracellular matrix components, including collagen and fibronectin, in addition to producing MMPs that degrade the matrix (29). Breakdown of the ECM releases embedded growth factors, including VEGF, thereby coupling matrix remodeling with neovascularization and tumor cell dissemination (30). At the same time, increased matrix stiffness and crosslinking, partly mediated by lysyl oxidase (LOX), activate integrin–FAK signaling and mechanoresponsive transcription programs such as YAP and TAZ, reinforcing a fibrotic and immune-excluding niche (31, 32). This dense stroma limits cytotoxic T-cell infiltration and alters dendritic cell trafficking, thereby promoting immune escape in EC (29, 33). Targeting ECM crosslinking, FAK signaling, or TGF-β–driven fibrosis may help normalize stromal architecture and enhance immune accessibility, though optimal combinations and predictive biomarkers remain to be defined (34–36).

#### 2.2.3 Estrogen signaling–immune crosstalk

The pathogenesis of endometrial cancer is strongly influenced by estrogen, which governs transcriptional regulation and orchestrates immune-related inflammatory responses (37). Through its activation of peritoneal macrophages, estrogen enhances the secretion of inflammatory mediators such as TNF-α and IL-1β. These cytokines subsequently stimulate the NF-κB pathway, promoting an inflammatory milieu that facilitates tumor advancement (38, 39). In EC tissues, an inverse correlation has been observed between TAMs infiltration and ERα expression. Specifically, TAM-derived CXCL8 suppresses ERα expression through HOXB13 induction, thereby enhancing tumor invasiveness (40). Estrogen also contributes to tumor progression by regulating immune-related genes such as ZNF626, SLK, and RFWD3, which influence the immune microenvironment (41). Collectively, the findings reveal that estrogen contributes to tumor progression through two distinct mechanisms: directly enhancing oncogenic gene expression and indirectly promoting immune escape by altering inflammation-associated immune dynamics.

#### 2.2.4 Obesity and metabolic inflammation

EC’s risk and progression are markedly influenced by obesity, largely due to sustained metabolic disturbances and chronic inflammation of low intensity (42, 43). Obesity is frequently linked with adipose tissue that exhibits hypoxic environments and the demise of adipocytes, subsequently prompting the release of inflammatory mediators, including TNF-α, IL-6, and MCP-1. These cytokines initiate NF-κB pathway activation, fostering a cellular environment that enhances proliferation, motility, and invasive capacity of endometrial cells, while concurrently suppressing programmed cell death (42, 43). Obesity also increases aromatase activity in adipose depots, resulting in elevated local estrogen production and amplification of estrogen-mediated proliferative and immunomodulatory effects (44). In addition, obesity-related gut dysbiosis can exacerbate systemic and local inflammation, partly through altered bile acid metabolism and disruption of farnesoid X receptor (FXR) signaling, thereby contributing to a pro-tumorigenic microenvironment (43).

#### 2.2.5 Endothelial activation and sterile inflammation

During EC progression, estrogen-mediated activation of endothelial cells is associated with elevated expression of inflammatory chemokines such as CXCL10, CXCL13, and IGF1, collectively contributing to a proinflammatory microenvironment (45). In mouse models of endometrial hyperplasia, increased levels of IL-1β and TNF-α, together with enhanced macrophage infiltration, indicate the presence of sterile inflammation within endometrial tissue (46). Notably, this inflammatory state may be sustained through bidirectional interactions between activated endothelial cells and infiltrating macrophages, independent of continuous estrogen stimulation (45). The findings underscore the role of endothelial activation as a significant enhancer of immune signaling in the surrounding tissue, which perpetuates a persistent inflammatory environment conducive to the initiation and advancement of endometrial cancer. Thus, disrupting endothelial-dependent inflammatory circuits could provide an effective strategy to reshape the immunological landscape of the tumor microenvironment.

## 3 Immune escape mechanisms in EC

### 3.1 Immune checkpoint pathways

As endometrial tumors evolve, the activation of immune checkpoint signaling becomes a central mechanism by which malignant cells escape immune detection and suppress cytotoxic responses. The immunological landscape of endometrial cancer is profoundly altered by the overexpression of multiple immune checkpoint regulators, including PD-L1, CTLA-4, TIM-3, and LAG-3. These molecules facilitate tumor immune evasion by inhibiting T cell responses via the engagement of inhibitory receptors (47). Specifically, when PD-L1 binds to PD-1 on T cells, it suppresses their proliferation and cytolytic function. In parallel, the association of CTLA-4 with its ligands CD80 and CD86 obstructs the initiation of T cell activation (47). Although checkpoint blockade therapies have demonstrated notable success in treating other cancer types, their effectiveness in endometrial cancer has been relatively limited. Elevated levels of PD-L1 expression have been associated with more advanced stages of the disease and poorer clinical outcomes, thereby underscoring the significance of immune checkpoints in forming an immunosuppressive tumor microenvironment (47). Of note, IL-17/Th17 signaling has been implicated in both up- and down-stream regulation of PD-L1 and antigen-presentation programs, with conflicting findings across molecular subtypes and cytokine milieus (48, 49). The unforeseen function of Th17/IL-17 extends to checkpoint regulation, where IL-17 amplifies PD-L1 expression under certain conditions and increases antigen presentation in others, depending on the surrounding cytokine milieu and molecular subtype.

In EC, persistent inflammation within the tumor microenvironment significantly affects the modulation of immune checkpoint ligands and their corresponding receptors. The stimulation of proinflammatory signaling pathways, particularly the NF-κB pathway, leads to an upregulation of crucial checkpoint molecules. Notably, cytokines such as TNF-α and IL-1β have been shown to increase PD-L1 expression via NF-κB-dependent mechanisms, thereby facilitating immune escape. Moreover, immune cells involved in inflammation, including dendritic cells and macrophages, release cytokines that further modulate checkpoint activity, thereby promoting tumor cell proliferation and dissemination. This cytokine-driven amplification of checkpoint signaling reinforces immune evasion. These findings underscore the promising therapeutic prospects of integrating immune checkpoint inhibitors with strategies aimed at modulating the inflammatory microenvironment, thereby enhancing treatment effectiveness in EC (13).

### 3.2 Impaired antigen presentation

Endometrial cancer cells frequently evade immune surveillance by reducing the expression of MHC class I (HLA class I) molecules, which play a vital role in presenting endogenous peptide antigens to cytotoxic T lymphocytes. When MHC I is downregulated, antigen visibility to cytotoxic T lymphocytes (CTLs) is diminished, allowing tumor cells to avoid immune-mediated destruction (16). This immune escape is often driven by deficient β2-microglobulin (B2M) expression, a protein indispensable for MHC I stability and trafficking to the cell surface (50). Interestingly, even with reduced MHC I expression, EC cells can avoid NK cell lysis through alternative mechanisms—such as upregulating non-classical HLA-E and HLA-G, shedding ligands that bind NKG2D receptors, or releasing immunosuppressive cytokines (51, 52). Clinically, diminished MHC I or B2M levels have been strongly correlated with later-stage disease, increased invasiveness, and a greater likelihood of metastasis (16). These findings identify impaired antigen presentation as both a hallmark of immune escape and a potential therapeutic target. Strategies aimed at restoring MHC I expression, stabilizing B2M, or integrating CTL- and NK cell–based approaches may enhance antitumor immunity in EC (53).

### 3.3 Metabolic immunosuppression

EC leverages multiple metabolic pathways to promote immune escape. Enhanced glycolysis leads to lactate accumulation, acidifying the tumor microenvironment and impairing effector T cells and NK cells, while also promoting M2 macrophage polarization via HIF-1α and MCT transporters. Overexpression of IDO1 depletes tryptophan and accumulates kynurenine, directly suppressing T-cell proliferation and altering immune infiltration, with elevated IDO1 levels correlating with advanced EC and poor prognosis (54–56). Systemic metabolic dysfunction, common in obese EC patients, further supports immunosuppression via insulin/IGF–PI3K–AKT–mTOR signaling and enhanced lipid metabolism in regulatory immune subsets. Additionally, metabolic byproducts such as lactate, adenosine, and PGE2 can upregulate immune checkpoints like PD-L1 through HIF-1α, NF-κB, and STAT3 pathways. These interconnected mechanisms reveal the therapeutic promise of integrating metabolic intervention with immune checkpoint blockade in EC.

### 3.4 Innate immune escape

Innate immune dysfunction is a key feature of immune escape in EC. NK cells often display reduced numbers, downregulated activating receptors (e.g., NKG2D, NKp30), and diminished cytolytic function, driven by tumor-derived cytokines, lactate, adenosine, and inhibitory checkpoints such as NKG2A and TIGIT (57, 58). Cytokines originating from tumors, including G-CSF, GM-CSF, IL-6, and IL-1β, play a significant role in promoting the proliferation of myeloid-derived suppressor cells (MDSCs), which are crucial for attenuating antitumor immune responses. MDSCs exert their immunosuppressive effects on T lymphocytes and NK cells through the expression of various immunosuppressive molecules such as ARG1, iNOS, and PD-L1. Additionally, they secrete reactive oxygen species and other soluble factors that further impede the activity of immune cells (59). These immunosuppressive populations, in concert with TAMs, Tregs, and stromal components, form a self-reinforcing inhibitory network that undermines checkpoint blockade efficacy. Therapeutic strategies under investigation include NK cell activation or adoptive transfer, MDSC depletion or reprogramming (e.g., via CXCR2, CSF1R, STAT3 inhibitors, or ATRA), and metabolic or vascular normalization to restore innate antitumor immunity (60–62).

## 4 Inflammatory microenvironment remodeling drives immune escape and therapy resistance in EC

### 4.1 Interaction between inflammatory microenvironment remodeling and immune escape

The dynamic interplay between inflammatory microenvironment remodeling and immune escape is critical in the progression of EC. Elevated levels of inflammatory cytokines have been shown to upregulate immune checkpoint molecules on tumor cells, thereby facilitating immune escape. Simultaneously, cytokines secreted by immunosuppressive cells such as TAMs and Tregs suppress antitumor immune responses, further enhancing the ability of cancer cells to evade immune surveillance (63). Previous studies have suggested that combining immune checkpoint inhibitors with anti-inflammatory agents may represent a promising therapeutic strategy for EC. This dual-targeting approach, which simultaneously addresses both the inflammatory microenvironment and tumor immune escape mechanisms, may yield synergistic effects and improve clinical outcomes (64). Moreover, therapies aimed at modulating TAMs and Tregs could reshape the tumor’s inflammatory milieu and enhance host antitumor immunity, offering novel avenues for EC treatment (64). Future research should elucidate the molecular crosstalk between inflammation and immune escape, enabling the discovery of novel targets and the advancement of precision immunotherapies in endometrial cancer.

### 4.2 Molecular mechanisms of IME remodeling drives therapy resistance in EC

Therapy resistance in EC stems from both tumor-intrinsic changes and inflammatory immune microenvironment (IME) remodeling. Persistent activation of pathways such as IL-6/JAK–STAT3 and TNF-α/NF-κB promotes anti-apoptotic signaling, immune checkpoint upregulation, and metabolic reprogramming, driving resistance to chemo-, radio-, and immunotherapy (65). Hypoxia-induced HIF-1α stabilization further reinforces immunosuppression by enhancing PD-L1 expression and glycolytic metabolism (65, 66). In parallel, stress-adaptive processes such as autophagy contribute to treatment tolerance. For example, kinase inhibitors have been shown to activate cytoprotective autophagy through the MAPK/JNK signaling axis, as demonstrated with agents like sorafenib (66, 67). Moreover, genetic alterations such as loss of ARID1A disrupt the SWI/SNF chromatin remodeling complex, leading to transcriptional reprogramming, impaired antigen presentation, and reduced therapeutic responsiveness (68). Together, these extrinsic and intrinsic mechanisms shape a multifaceted resistance phenotype in EC, underscoring the need for combination strategies that co-target inflammation, immune suppression, and tumor cell plasticity.

## 5 Therapeutic targets and strategies in EC

### 5.1 Treatment targeting IME and their signaling pathways

Therapeutic strategies targeting the inflammatory microenvironment aim to inhibit inflammatory mediator secretion and to modulate immune cell infiltration and polarization. Cyclooxygenase-2 (COX-2) inhibitors reduce proinflammatory mediators and can ameliorate the inflammatory milieu (69). Regulation of macrophage polarization can shift protumorigenic M2-like TAMs toward an antitumorigenic M1-like phenotype (21). IME-directed approaches have shown efficacy in multiple tumors (70), but their application in EC remains exploratory, and further studies are needed to define mechanisms and clinical benefit in EC.

The inflammatory microenvironment (IME) in endometrial cancer is coordinated by converging pathways that promote progression and immune escape. Toll-like receptor 4 (TLR4) signaling enhances proinflammatory cytokine production through NF-κB activation, shaping a tumor-permissive milieu (71). The COX-2–PGE2 axis supports carcinogenesis by driving proliferation and suppressing antitumor immunity; combined targeting of COX-2 and EP receptors has shown therapeutic potential (72, 73). Wnt/β-catenin signaling intersects with inflammatory networks, contributing to immune exclusion and cancer stemness (74, 75). NF-κB, often activated by TNF-α in obesity-related EC, also regulates GLUT6, linking inflammation to metabolic reprogramming (76). Together, these pathways sustain an immunosuppressive and therapy-resistant niche, underscoring their value as targets for IME-directed therapies.

### 5.2 Treatment targeting immune escape

Surgical resection remains the primary treatment for EC, with radiotherapy and chemotherapy frequently used as adjunctive modalities (77). However, for patients with advanced, metastatic, or recurrent EC, effective therapeutic options remain limited. This highlights the need for novel strategies to improve prognosis. Immunotherapy, a major advance in oncology, has increasingly become a focus of research and offers promise for EC treatment (78).

To counteract immune escape in endometrial cancer, current treatment modalities incorporate immune checkpoint inhibitors and CAR-T cell therapies. These agents reinvigorate impaired T cell responses by disrupting immunosuppressive pathways, notably those mediated by PD-1/PD-L1 and CTLA-4 interactions (2). CAR-T cell therapy, by contrast, involves the genetic modification of T cells to confer specificity against tumor-associated antigens, enabling direct recognition and elimination of malignant cells. Emerging clinical evidence supports the notion that integrating checkpoint inhibitors with chemotherapy or radiotherapy can significantly enhance therapeutic efficacy in EC patients (79). This integrated strategy not only amplifies antitumor immunity by activating multiple pathways but also overcomes some limitations of monotherapies, providing a more comprehensive therapeutic approach (79). Ongoing research should focus on identifying optimal combination regimens to offer more effective treatment options for EC patients.

### 5.3 Clinical landscape of IME-targeted therapies in EC

Targeting the IME has become central to EC therapy, as immune cells, stromal components, and cytokine networks drive progression, immune escape, and resistance (80). Combining immunotherapy with microenvironment modulation shows clinical promise. The KEYNOTE-775 study, assessing participants from both pMMR and dMMR groups, found that combining pembrolizumab with lenvatinib markedly enhanced survival without disease progression (7.2 vs. 3.8 months) alongside total survival rates (18.3 vs. 11.4 months) in contrast to the physician’s selection between doxorubicin and paclitaxel (81). In mismatch repair-deficient (dMMR) or microsatellite instability-high (MSI-H) EC, the GARNET trial supported FDA approval of dostarlimab monotherapy, which achieved a 45.5% response rate with durable benefit (81, 82). Immune profiling is emerging as a predictor of therapeutic response. Clinical data from a multicenter trial indicate that patients receiving the combination of pembrolizumab and lenvatinib benefit from enhanced therapeutic outcomes, particularly in cases with substantial CD20^+^ B-cell infiltration and a high CD8/CD20 ratio within the tumor microenvironment (83, 84). Likewise, endometrial tumors harboring p53 mutations, enriched in tumor-infiltrating lymphocytes (TILs) and expressing high levels of immune evasion molecules, appear to be especially responsive to combined treatment strategies involving checkpoint inhibitors and precision-targeted therapies, including those directed at PARP or HER2 pathways (85). Stromal remodeling also contributes to resistance. Although genetically stable, stromal cells can be reprogrammed by tumor-derived signals to promote immune suppression and metastasis, underscoring the therapeutic potential of targeting tumor–stroma crosstalk (86–88). Together, these insights support a precision framework that integrates immune and stromal profiling with IME-targeted therapies to optimize outcomes in EC. Together, these insights support a precision framework that integrates immune and stromal profiling with IME-targeted therapies to optimize outcomes in EC. **Table 1** summarize pivotal randomized and registration trials across first- and later-line settings, stratified by dMMR/MSI-H versus MSS/pMMR and listing key endpoints (OS, PFS, ORR).

**Table 1:** Key clinical trials of immunotherapy and targeted regimens in endometrial cancer.

NCT number	Trial	Phase	Line	Patients (n)	Population	Regimen	Comparator	mOS (mo) [HR (95% CI), p]	mPFS (mo) [HR (95% CI), p]	ORR (%) [95% CI]
NCT03517449	KEYNOTE-775/Study 309	III	Second line	827	All-comers (pMMR + dMMR)	Lenvatinib + Pembrolizumab	Doxorubicin or Paclitaxel	18.3 vs 11.4 [HR 0.62]	7.2 vs 3.8 [HR 0.56]	31.9 vs 14.7
NCT03914612	NRG-GY018/KEYNOTE-868	III	First line	810	pMMR + dMMR (stratified)	Pembrolizumab + Carboplatin/Paclitaxel → Pembrolizumab maintenance	Placebo + Carboplatin/Paclitaxel → Placebo	NR (interim)	PFS HR 0.30 (dMMR); 0.57 (pMMR)	—
NCT03981796	RUBY (Part 1)	III	First line	494	Overall; dMMR subgroup prespecified	Dostarlimab + Carboplatin/Paclitaxel → Dostarlimab maintenance	Placebo + Carboplatin/Paclitaxel → Placebo	24-mo OS 71.3% vs 56.0 [HR 0.64]	Overall HR 0.64; dMMR HR 0.28	—
NCT04269200	DUO-E	III	First line	718	Overall (maintenance randomization)	Durvalumab + Carboplatin/Paclitaxel → Durvalumab ± Olaparib	Placebo + Carboplatin/Paclitaxel → Placebo	Interim: HR 0.77 (Durva); 0.59 (Durva+Olap)	HR 0.71 (Durva); 0.55 (Durva+Olap)	—
NCT02715284	GARNET	I/II (single-arm)	≥2L	—	dMMR/MSI-H EC	Dostarlimab	—	—	—	45.5 [95% CI ~37–54]
NCT02628067	KEYNOTE-158 (EC cohort)	II (single-arm)	≥2L	—	MSI-H/dMMR EC	Pembrolizumab	—	—	—	~46 [~35–56]
NCT01367002	Trastuzumab + Chemo in HER2+ USC	II	First line/Recurrent	61	HER2-positive uterine serous carcinoma	Trastuzumab + Carboplatin/Paclitaxel	Carboplatin/Paclitaxel	29.6 vs 24.4 [HR 0.58]	12.6 vs 8.0 [HR 0.44]	—

Carbo, carboplatin; dMMR, mismatch-repair deficient; pMMR, mismatch-repair proficient; Durva, durvalumab; Olap, olaparib; EC, endometrial cancer; OS, overall survival; PFS, progression-free survival; ORR, objective response rate; NR, not reached. Notes: Data shown reflect primary manuscripts and interim analyses where indicated. For NRG-GY018 and RUBY, hazard ratios are presented for prespecified dMMR and pMMR cohorts.

## 6 Conclusion and prospect

The inflammatory microenvironment (IME) is a key driver of EC initiation, progression, and therapy resistance. Dysregulated inflammatory signaling and immune escape mechanisms—mediated by immune, stromal, and metabolic components—collectively shape an immunosuppressive niche and poor clinical outcomes. Recent developments in single-cell and spatial technologies have illuminated the diversity and adaptability of IME populations, highlighting the necessity for targeted approaches that consider their evolving nature. Therapeutic approaches targeting cytokine networks, immunosuppressive mediators, or metabolic checkpoints, as well as functional reprogramming of immune and stromal cells, are under active investigation. Combining IME-directed interventions with immune checkpoint inhibitors or anti-angiogenic agents shows synergistic potential, though challenges remain due to IME heterogeneity, context-dependent functions, and the lack of predictive biomarkers. Future research integrating multi-omics profiling and spatial mapping will be crucial for identifying molecular signatures of response and resistance, enabling patient stratification and precision immunotherapy. Ultimately, disrupting the cycle of inflammation, immune escape, and resistance may transform the IME from a barrier into a therapeutic opportunity in EC management.
